# Was It Pulmonary Embolism?

**Published:** 1887-10

**Authors:** A. J. Gray

**Affiliations:** Martin’s Mills


### WAS IT PULMONARY EMBOLISM?

*By A. J. Gray, M. D., Martin's Mills.*

Read before Vanzandt County Medical Society, and Contributed to Daniel's Texas Medical Journal.

I WAS called on March 26, 1886, in consultation with Dr. S., to see Mrs. B., a young woman in the eighth month of utero-gestation. She was suffering with severe pain in the right side, which seemed to be produced from encroachment of the child on the diaphragm, for a few small doses of morphine gave relief. The patient was a subject of marked blood discrasia, having had several attacks of intermittent fever since the time of conception. There was deep palor, with bloated face and extremities. She had sore mouth and throat, which answered in every particular to nurses sore mouth; she was very weak, and anorexia was complete, for if any food was forced on the stomach it was soon ejected. A speculum examination revealed ulceration of the neck of the womb. We made an application of nitrate silver to ulcers, and ordered to be given iron, wine, and all the nourishing food the stomach would retain. On the next day patient was some better; rested well through the night, and was able to take and retain some food. The same treatment was continued, and I did not see the patient any more till the 30th, four days after I was first called.

I was sent for in haste on the morning of the 30th, and arriving at 1 o'clock p. m., found the lady in labor. Pains commenced at 10 o'clock a. m., were regular, with some increase in force. At 4 o'clock p. m. I made examination, and found head presentation, first position, according to Playfair. Dr. S. not being present, I had him sent for. Labor continued to increase, and at 1 o'clock a. m. of 31st she was delivered of a dead child, labor having been natural throughout, She was delivered of the afterbirth in a few minutes without the loss of any blood, and after resting for a short while, she expressed herself as feeling better than for many days past. After waiting and watching for an hour and thirty minutes, and finding that the womb was well contracted, and that nothing was present to indicate immediate danger, Dr. S. left for home and I retired to rest for a short time.

At 4 o'clock I was aroused by the husband, who informed me that his wife was not resting well. I immediately went to her bedside, and found her restless, anxious, and breathing heavily; pulse 120, temperature normal. I diagnosed pulmonary obstruction, and immediately went to work to obviate the danger. I ordered plenty of whisky, carb. ammonia and digitalis, hoping that life might be prolonged until the obstruction could be absorbed, but all was without avail, for she continued to grow worse every minute; the dyspnœa increased in intensity, and became most horrible; she gasped and struggled for breath, and would make frequent efforts to tear off the covering from her chest in the effort to get more air. The muscles of the face and thorax were violently strained in the vain attempt to oxygenate the blood, and at times she was threatened with convulsions. The face was pale, and lips purple; the countenance expressed deep anxiety. The circulation was irregular; the action of the heart tumultuous, and it became more and more feeble and fluttering; the respirations grew shorter and more hurried. The intellect throughout the struggle was unimpaired, and the dreadful consciousness of impending death added no little to the terror of the scene. At 10 o'clock a. m., about six hours from the first beginning of the dyspnœa, death ended the struggle.

Now, I believe this to have been a case of pulmonary obstruction, of spontaneous origin, commencing in the smaller ramifications of the pulmonary arteries, and gradually extending back toward the heart, a condition which seems to me might occur more frequently, when we take into consideration the condition of the patient referred to, she being a subject of marked blood dyscrasia, then just passed through labor and entered the puerperal state, where we find most of the conditions favoring thrombosis present; the blood contains at that time an excess of fibrin, which increases in the last months of utero-gestation. The involution of the enlarged womb commences, and the blood is loaded wlth effete material, which further increases its depravity, and brings about so many of the conditions favoring thrombosis.

Playfair tells us that the attention of the profession has been for the most part limited to a study of one only of the results of this tendency to blood clotting, after delivery, no doubt, because of its comparative frequency and evident symptoms. True, it has of late been held that phlegmasia-dolens is chiefly the result of some morbid condition of the blood, producing plugging of the veins, but the view which I am attempting to express would bring this disease into close relation with the more rarely observed, but infinitely important condition—obstruction of the pulmonary arteries, a result or accident that has not received that attention it really deserves, for in the present state of our knowledge of its pathology and treatment, it swoops down upon us like the eagle on his prey, and seldom fails to carry off our patient.

Now, if this paper succeeds in bringing before the minds of the profession of Vanzandt county a study of this important disease, it will have served its purpose.

